# Adolescent BMI and early-onset type 2 diabetes among Ethiopian immigrants and their descendants: a nationwide study

**DOI:** 10.1186/s12933-020-01143-z

**Published:** 2020-10-06

**Authors:** Maya Simchoni, Uri Hamiel, Orit Pinhas-Hamiel, Inbar Zucker, Tali Cukierman-Yaffe, Miri Lutski, Estela Derazne, Zivan Beer, Doron Behar, Lital Keinan-Boker, Ofri Mosenzon, Dorit Tzur, Arnon Afek, Amir Tirosh, Itamar Raz, Gilad Twig

**Affiliations:** 1 Department of Military Medicine, Hebrew University, Jerusalem and the Israel Defense Forces Medical Corps, Ramat Gan, Israel; 2 Genetic Institute, Tel Aviv Sourasky Medical Center, Tel Aviv, Israel; 3 Sackler Faculty of Medicine, Tel Aviv University, Tel Aviv, Israel; 4 Department of Pediatric Endocrinology, Safra Children Hospital, Sheba Medical Center, Tel Hashomer, Ramat Gan, Israel; 5 The Israel Center for Disease Control, Ministry of Health, Ramat Gan, Israel; 6 Institute of Endocrinology, Sheba Medical Center, Tel Hashomer, Ramat Gan, Israel; 7 Igentify, Tirat Hacarmel, Israel; 8 The Diabetes Unit, Department of Internal Medicine, Hadassah Hebrew University Hospital, Jerusalem, Israel; 9 Central Management, Sheba Medical Center, Tel Hashomer, Ramat Gan, Israel; 10 Department of Epidemiology and Preventive Medicine, School of Public Health, Sackler Faculty of Medicine, Tel Aviv University, Tel Aviv, Israel

**Keywords:** Adolescents, BMI, Early-onset, Epidemiology, Immigration, Type 2 diabetes

## Abstract

**Background:**

We assessed in a nationwide cohort the association between adolescent BMI and early-onset (< 40 years) type 2 diabetes among Israelis of Ethiopian origin.

**Methods:**

Normoglycemic adolescents (range 16–20 years old), including 93,806 native Israelis (≥ 3rd generation in Israel) and 27,684 Israelis of Ethiopian origin, were medically assessed for military service between 1996 and 2011. Weight and height were measured. Data were linked to the Israeli National Diabetes Registry. Incident type 2 diabetes by December 31, 2016 was the outcome. Cox regression models stratified by sex and BMI categories were applied.

**Results:**

226 (0.29%) men and 79 (0.18%) women developed diabetes during 992,980 and 530,814 person-years follow-up, respectively, at a mean age of 30.4 and 27.4 years, respectively. Among native Israeli men with normal and high (overweight and obese) BMI, diabetes incidence was 9.5 and 62.0 (per 10^5^ person-years), respectively. The respective incidences were 46.9 and 112.3 among men of Ethiopian origin. After adjustment for sociodemographic confounders, the hazard ratios for type 2 diabetes among Ethiopian men with normal and high BMI were 3.4 (2.3–5.1) and 15.8 (8.3–30.3) respectively, compared to third-generation Israelis with normal BMI. When this analysis was limited to Israeli-born Ethiopian men, the hazard ratios were 4.4 (1.7–11.4) and 29.1 (12.9–70.6), respectively. Results persisted when immigrants of other white Caucasian origin were the reference; and among women with normal, but not high, BMI.

**Conclusions:**

Ethiopian origin is a risk factor for early-onset type 2 diabetes among young men at any BMI, and may require selective interventions.

## Background

Diabetes mellitus is a major cause of morbidity and mortality. The global prevalence of type 2 diabetes among adults over age 18 years has exceeded 8.5%. Over 468 million are estimated to be affected [1]; the mean age of diagnosis has decreased over the last couple decades [2]. Early-onset (< age 40 years) type 2 diabetes is associated with a worse clinical course and premature development of complications [3]. Several risk factors are associated with early-onset diabetes, among them adolescent obesity, certain ethnic backgrounds, immigration, and lower socioeconomic position. Additionally, there are some discrepancies between adults with type 2 diabetes from different ethnicities and risk of cardiovascular morbidity [4, 5].

Severe obesity in childhood is a risk factor for cardiovascular morbidity [6], and among children in the US, BMI at baseline preceded higher insulin levels at follow-up during childhood, with blacks and whites showing similar patterns of this one-directional relationship [7]. Immigrants from low- and middle-income countries including those of African and other ancestries have been shown to have a disproportionately high risk for type 2 diabetes, which may be evident at a lower body-mass index (BMI) [8]. Among African immigrants to the US [8, 9] and to Europe [10] significantly higher risk for pre-diabetes and diabetes have been shown, compared to the native populations. Nevertheless, evidence among adolescent immigrants from Africa is limited, as this population is underrepresented in epidemiological studies and is frequently considered a homogenous group despite large ethnic variability [11, 12].

As a destination for immigration from several regions worldwide, including Ethiopia, Israel provides an opportunity to address the risk of diabetes among persons of East African origin. Moreover, systematic documentation is available of adolescent health status for over five decades. Here we assessed the risk for early-onset type 2 diabetes among Israeli adolescents of Ethiopian origin compared to native Israelis and compared to other immigrant populations to Israel. Our goal was to assess whether the incidence of early-onset type 2 diabetes is higher in Israelis of Ethiopian origin than among native Israelis, and to specifically assess whether this relation differs according to adolescent BMI, sex, and immigration status.

## Materials and methods

### Study population

This was a retrospective cohort study. At age 17 years, Israeli adolescents are approached to undergo a medical evaluation prior to mandatory military service. The study sample comprised all Israeli male and female adolescents who were medically evaluated for military service between ages 16–20 years from January 1, 1996 for men, and January 1, 1997 for women, until December 31, 2011. We excluded examinees with missing BMI data (n = 3,291, 2.6%) and those with a history of diabetes or dysglycemia at study entry (n = 325, 0.3%) that was solely based on reports by primary care physicians. We also excluded those who died before the establishment of the Israel National Diabetes Registry (INDR) in 2012 (n = 378; 0.3%) as these individuals could not be reported to the registry (See Additional file 1: Figure S1). The study sample included 121,490 examinees for whom we had continuous follow-up from adolescence until diabetes onset, death, or December 31st, 2016, whichever came first. Data of an additional 121,997 male adolescents who immigrated to Israel from former countries of the Union of Soviet Socialist Republics (USSR) during the same years as the immigration from Ethiopia were analyzed as part of sensitivity analyses. The Israel Defense Forces Medical Corps Institutional Review Board approved this study.

### The Israel National Diabetes Registry (INDR) and diagnosis of diabetes

The primary outcome of the study was incident type 2 diabetes that was diagnosed either during military service, or later in life as recorded by the INDR. All health medical organizations in Israel have been requested by law to annually report prevalent cases of diabetes to the INDR since 2012. These organizations provide medical care to nearly 100% of permanent residents in Israel. The INDR retrospectively recorded the date of diabetes diagnosis for all individuals diagnosed in Israel between January 1, 2000 and December 31, 2011, and prospectively recorded all new incidences of diabetes from January 1, 2012 onwards. Data of the INDR were linked to the IDF database using the participants’ national ID number, to enable the linkage of body weight and height measured at adolescence with diabetes incidence recorded later in life. Diabetes was reported to the INDR when one or more of the following criteria were met in the previous year of the report to the registry: (i) glycated hemoglobin (HbA1c) ≥ 6.5%; (ii) serum glucose concentrations of ≥ 200 mg/dL in two tests performed at an interval of at least one month; (iii) ≥ 3 purchases of glucose lowering medications in different months. Data regarding prescribed diabetes medications were used to classify diabetes as type 1, type 2, or uncertain type, as reported previously [13] (for details see the Additional file 1: Appendix). The sensitivity of the INDR for the detection of type 2 diabetes is 95% [13] The INDR also includes weight and height measurements that were documented at routine clinic visits during the year of diabetes diagnosis. Measurements regarding weight and height at adulthood among those who did not develop diabetes were unavailable. Individuals who were reported to the INDR as having type 2 diabetes, but without a formal date of diagnosis were considered missing in the time-to-event analysis if they were first reported in the first year of the registry, 2012; and were included only in logistic regression models. For individuals without a formal date, who were first reported in 2013 or later, we assigned the date of diagnosis as July 1^st^ of the year before the report year to the INDR, as described previously [14].

### Data collection and study variables

Age at examination and year of birth were treated as continuous variables. The health examination was performed by military physicians who reviewed the participants’ medical records and provided diagnostic codes when applicable. The physical examination included measurements of weight and height (barefoot and in underwear) by trained medics using a beam balance and stadiometer. Measurements were recorded and rounded to the nearest 0.5 kg for weight and one cm for height. BMI was calculated (weight in kilograms divided by height squared in meters) and categorized into 3 subgroups: < 18.5 kg/m^2^ (underweight), 18.5–24.9 kg/m^2^ (normal BMI), and ≥ 25 kg/m^2^ (high BMI)*.* Because at age 17 adolescents have completed > 98% of their growth potential, we used the world health organization (WHO) BMI categories intended for adults [15]. Individuals whose medical assessment revealed no history of major operation or comorbidity and who had no illness that requires a chronic medical treatment were defined as having unimpaired health. Data regarding education and residential socioeconomic status (SES) were obtained from governmental ministries, and were used as previously reported [16]; details are provided in the Additional file 1: Appendix. Cognitive performance at adolescence was assessed by a general intelligence test that was delivered to all individuals at study entry, and was categorized (low/medium/high) as reported previously [17]. Notably, this variable was identified as an independent risk factor for early-onset type 2 diabetes in a subpopulation of this cohort [18]. Examinees were classified as of Ethiopian origin if they or their fathers were born in Ethiopia, whereas Israeli-born individuals whose fathers and grandfathers were born in Israel were classified as native Israelis [19]. All individuals of Ethiopian origin were either immigrants from Ethiopia or were born in Israel to immigrants from Ethiopia (second-generation). In a similar manner, we defined in a sensitivity analysis the region of origin of immigrants or offspring of immigrants from former countries of the USSR [20].

### Statistical analysis

Prior to undertaking analysis, due to clinical plausibility, we decided to stratify the cohort by sex and adolescent BMI (underweight, normal, and high BMI). Analysis of the entire cohort demonstrated a significant interaction term between sex and origin (p = 0.038), and a borderline significant interaction term between BMI and origin (p = 0.086).

The incident rate of type 2 diabetes was calculated per person-year of follow-up for native Israelis and Israelis of Ethiopian origin per a given BMI group. Kaplan–Meier survival curves were computed for the BMI percentile categories, with 95% confidence intervals. Cox proportional hazard models were used to estimate the hazard ratios (HRs) and 95%-confidence intervals (CIs) for incident diabetes, considering native Israelis with normal BMI as the reference group. Covariates were added in a stepwise manner to a model adjusted for age and birth year. Variables that were significant (p < 0.05) in the minimally adjusted model were included in the final multivariable analysis. The assumption of proportionality of hazards was visually confirmed for all variables.

We conducted several sensitivity analyses that were limited to men, given the smaller sample of women in this cohort. (i) The analysis was restricted to those with unimpaired health status at enrollment in order to minimize residual confounding by co-existing morbidities. (ii) The analysis was restricted to second-generation, Israeli-born men of Ethiopian origin, using the same reference group as the main analysis. (iii) We assessed the risk for type 2 diabetes that was related to age of immigration among immigrants from Ethiopia with normal adolescent BMI. (iv) Diabetes risk of men from former countries of the USSR were compared to the main study groups, since immigration to Israel from this region occurred in parallel to immigration from Ethiopia to Israel; 1990–2000 vs. 1985–2000. To better assess the contribution of immigration status to diabetes risk, analysis was stratified to immigrants (USSR immigrants were the reference) and second-generation Israeli-born (native Israelis were the reference). (v) Logistic regression models were applied to account for cases with missing date of type 2 diabetes diagnosis. (vi) We assessed potential misclassification of type 2 diabetes by accounting for cases of diabetes of uncertain type, or by using a stricter definition of type 2 diabetes with a positive predicted value of over 98% [13], in which a case of type 2 diabetes is defined only if reported to the INDR in two separate years.

## Results

Baseline characteristics of the men and women in this study are presented separately in Table 1. The age at study entry was similar across BMI groups in both sexes. Adolescents of Ethiopian origin of both sexes had lower residential SES than did native Israelis. Among Ethiopian Israelis of both sexes, most of those in the high BMI groups were born in Israel.

**Table 1:** Baseline characteristics of the study population according to origin and adolescent BMI groups

	Native Israelis			Ethiopian Israelis		
	Underweight BMI < 18.5	Normal BMI 18.5 ≤ BMI < 25	High BMI BMI ≥ 25	Underweight BMI < 18.5	Normal BMI 18.5 ≤ BMI < 25	High BMI BMI ≥ 25
*Men*						
Total number	7,435	42,600	10,736	5,307	10,708	1,035
Israeli-born (%)	100	100	100	32.5	24.6	59.7
Mean age (years ± SD)	17.3 ± 0.5	17.3 ± 0.5	17.4 ± 0.6	17.7 ± 0.7	17.8 ± 0.8	17.5 ± 0.6
Mean BMI (kg/m^2^ ± SD)	17.5 ± 0.8	21.3 ± 1.7	28.5 ± 3.3	17.3 ± 0.9	20.6 ± 1.6	28.4 ± 3.3
Mean weight (kg ± SD)	53.2 ± 4.8	64.7 ± 7.2	86.7 ± 12.3	51.0 ± 4.7	59.8 ± 6.6	84.8 ± 11.9
Mean height (cm ± SD)	173.9 ± 6.8	174.0 ± 6.7	174.1 ± 6.8	171.5 ± 6.8	170.3 ± 6.6	172.9 ± 7.1
Full education (%)	92.0	92.5	92.1	90.8	91.4	92.3
Low SES (%)	43.2	43.6	46.9	35.1	36.1	35.8
Medium SES (%)	35.2	35.4	36.2	62.0	60.4	61.1
High SES (%)	21.6	21.0	16.9	2.9	3.5	3.1
Low cognitive score (%)	23.2	20.6	25.1	65.6	63.3	64.5
Medium cognitive score (%)	62.2	64.1	63.5	34.2	36.3	34.8
High cognitive score (%)	14.2	15.4	11.4	0.2	0.3	0.7
*Women*						
Total number	4,685	23,170	5,180	3,114	6,507	1,013
Israeli-born (%)	100	100	100	36.5	34.5	51.6
Mean age (years ± SD)	17.1 ± 0.3	17.1 ± 0.3	17.2 ± 0.4	17.6 ± 0.7	17.6 ± 0.7	17.5 ± 0.7
Mean BMI (kg/m^2^ ± SD)	17.5 ± 0.8	21.3 ± 1.7	28.5 ± 3.4	17.2 ± 0.9	20.9 ± 1.7	28.1 ± 3.0
Mean weight (kg ± SD)	46.6 ± 4.1	56.0 ± 6.1	75.1 ± 10.9	44.3 ± 4.0	53.1 ± 5.8	72.3 ± 9.9
Mean height (cm ± SD)	163.0 ± 6.3	162.1 ± 6.1	162.2 ± 6.4	160.4 ± 6.0	159.1 ± 6.1	160.1 ± 6.7
Full education (%)	98.3	98.5	97.3	93.1	92.8	94.8
Low SES (%)	16.5	17.7	20.0	33.9	34.1	30.7
Medium SES (%)	49.3	49.8	52.8	64.2	62.8	65.5
High SES (%)	34.2	32.5	27.2	1.9	3.1	3.8
Low cognitive score (%)	14.6	12.7	18.3	62.1	62.5	60.7
Medium cognitive score (%)	73.6	74.3	72.5	37.6	37.2	38.5
High cognitive score (%)	11.8	13.0	9.2	0.3	0.3	0.8

BMI vales are presented in kg/m^2^ units. *SES* socioeconomic status (according to residence)

### Incident type 2 diabetes among Israeli men of Ethiopian Origin

During 992,980 person-years there were 226 incident cases of type 2 diabetes in men: 89 (0.52%) in those of Ethiopian origin and 137 (0.22%) in native Israelis. The mean ages at diabetes diagnosis were 30.3 and 30.4 years, respectively (p = 0.9). The crude incidence rates for type 2 diabetes (per 10^5^ person-years) in Ethiopian vs. native Israeli men were 17.3 vs. 5.1 in the underweight group, 46.9 vs. 9.5 in the normal BMI group, and 112.3 vs. 62 in the high BMI group (Table 2). The mean BMI values that were measured at the time when diabetes diagnoses were first reported to the INDR were lower in Ethiopian than native Israeli men for the normal and high adolescent BMI categories (Additional file 1: Figure S2A). Most native (69.4%), but not Ethiopian (49.0%), Israelis were with overweight or obesity at diabetes diagnosis (p = 5.2*10^–7^).

**Table 2:** Hazard ratios for type 2 diabetes incidence among Israelis of Ethiopian origin and native Israelis

	Native Israelis			Ethiopian Israelis		
	Underweight	Normal BMI	High BMI	Underweight	Normal BMI	High BMI
*Men*						
Number of diabetes incidences	5	52	80	12	65	12
Mean follow-up (years; ± SD)	13.3 ± 4.7	12.8 ± 4.6	12.1 ± 4.5	13.1 ± 4.6	13.0 ± 4.6	10.4 ± 3.8
Cumulative follow-up (person-years)	98,635.6	546,719.6	129,126.1	69,269.4	138,542.1	10,687.5
Incident rate (per 10^5^ person-years)	5.1	9.5	62.0	17.3	46.9	112.3
Mean age at diagnosis (years; ± SD)	28.7 ± 5.2	30.9 ± 4.6	30.2 ± 4.2	30.6 ± 2.9	30.8 ± 4.2	27.5 ± 3.5
HR (unadjusted)	0.51	Reference	7.24	1.78	4.88	17.93
95% CI	0.20–1.26		5.11–10.27	0.95–3.33	3.39–7.03	9.55–33.66
p-value	0.15		1.2 × 10^–28^	0.07	1.6 × 10^–17^	2.7 × 10^–19^
HR (adjusted*)	0.52	Reference	6.93	1.18	3.41	15.83
95% CI	0.21–1.31		4.86–9.88	0.60–2.29	2.29–5.06	8.29–30.25
p-value	0.17		1.3 × 10^–26^	0.63	1.4 × 10^–9^	6.3 × 10^–17^
*Men with unimpaired health*						
Number of new diabetes cases	3	36	53	9	44	11
HR (adjusted*)	0.46	Reference	6.91	1.26	3.04	20.15
95% CI	0.14–1.50		4.49–10.65	0.59–2.66	1.89–4.87	10.01–40.56
p-value	0.20		1.7 × 10^–18^	0.55	4.1 × 10^–6^	3.9 × 10^–17^
*Second-generation (Israeli-born) Ethiopians*						
Number of new diabetes incidences	5	52	80	2	5	6
HR (adjusted*)	0.52	Reference	6.96	2.25	4.42	29.10
95% CI	0.21–1.31		4.88–9.93	0.54–9.43	1.71–11.42	11.99–70.61
p-value	0.17		1.1 × 10^–26^	0.27	2.1 × 10^–3^	9.2 × 10^–14^
*Women*						
Number of diabetes incidences	0	16	34	4	19	6
Mean follow-up (years; ± SD)	12.4 ± 4.2	12.2 ± 4.3	11.8 ± 4.2	12.2 ± 4.1	12.3 ± 4.2	11.0 ± 3.9
Cumulative follow-up (person-years)	58,210.7	282,466.6	60,968.6	38,017.4	80,045.6	11,104.7
Incident rate (per 10^5^ person-years)	–	5.7	55.8	10.5	23.7	54.0
Mean age at diagnosis (years; ± SD)	–	26.1 ± 4.4	27.8 ± 3.7	24.6 ± 4.4	29.8 ± 4.0	26.1 ± 4.7
HR (unadjusted)	–	Reference	10.15	1.87	4.17	10.79
95% CI	–		5.60–18.39	0.62–5.59	2.15–8.12	4.22–27.60
p-value	–		2.1 × 10^–14^	0.26	2.6 × 1^–5^	6.9 × 10^–7^
HR (adjusted*)	–	Reference	9.66	1.13	2.40	7.04
95% CI	–		5.32–17.54	0.36–3.56	1.13–5.09	2.61–18.94
p-value	–		8.9 × 10^–14^	0.08	0.02	1.1 × 10^–4^

The table shows follow-up data, incident case data, and hazard ratios (HRs) in unadjusted and multivariable models (adjusted for birth year, age at study entry, education level, and cognitive score) in men and women. In men, two additional sub-analyses were done: HRs for individuals with unimpaired health and HRs for those Israeli-born (second-generation of immigrants)

*CI* confidence interval, *SD* standard deviation, *HR* hazard ratio

**Fig. 1:**
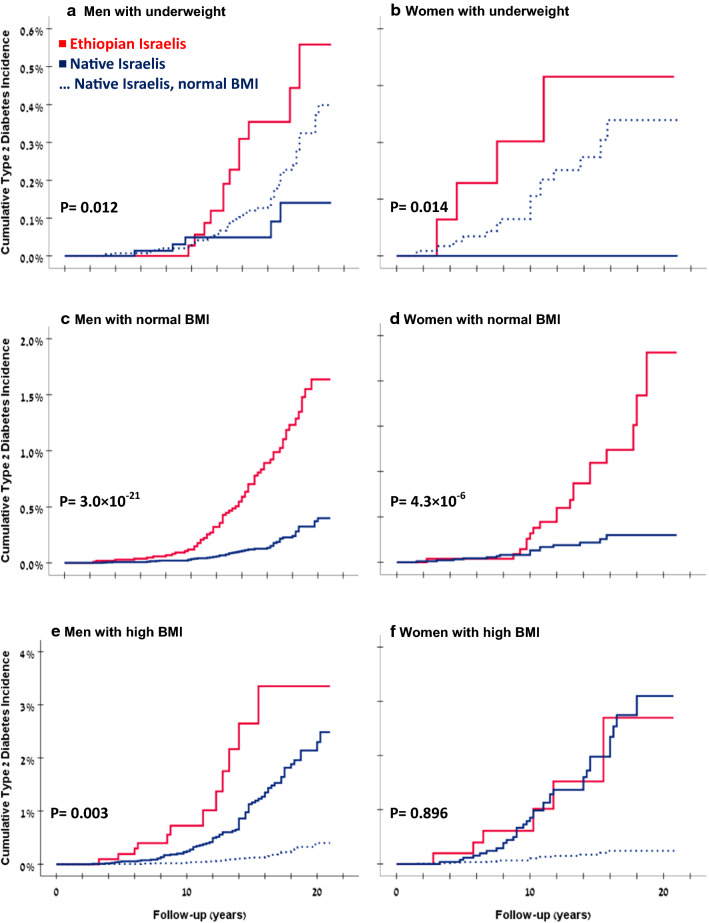
Associations between origin and cumulative type 2 diabetes incidence by adolescent BMI categories. Kaplan–Meier curves were plotted for each adolescent BMI category, for males and females separately. The dashed line represents the native Israelis with normal BMI group for each sex

Kaplan–Meier survival analysis curves for men are shown in Fig. 1a, c, e. The unadjusted HRs for type 2 diabetes were consistently higher in men of Ethiopian origin than in native Israeli men (Table 2). Further adjustment for sociodemographic data (education level, residential SES status, and cognitive performance) yielded the following HRs in Ethiopian vs. native Israelis (reference group, native Israelis with normal BMI): 1.2 (0.6–2.3) vs. 0.5 (0.2–1.3) in underweight, 3.4 (2.3–5.1) vs. 1 (reference) in normal BMI, and 15.8 (8.3–30.3) vs. 6.9 (4.9–9.9) in the high BMI group. The results persisted when the study sample was limited to persons with unimpaired health at baseline to minimize confounding by pre- or coexisting morbidities (Table 2).

**Fig. 2:**
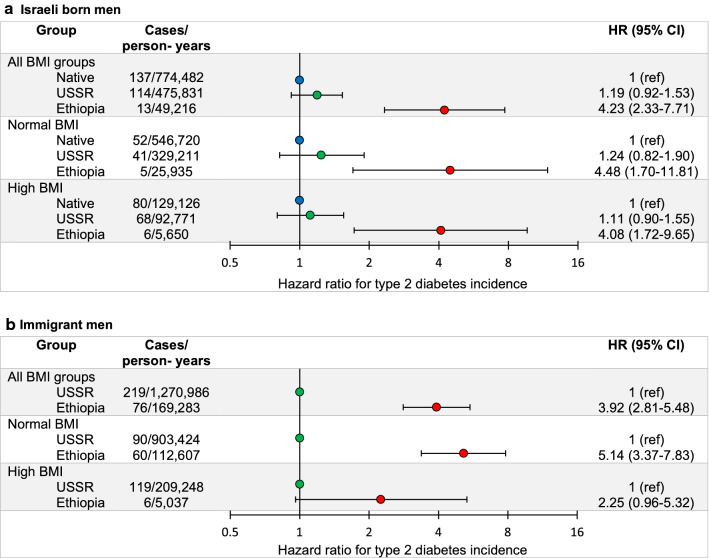
The association between male adolescent BMI and early onset type 2 diabetes among immigrants from another origin. This association was tested among immigrants from the Union of Soviet Socialist Republics (USSR), whose immigration to Israel paralleled the immigration from Ethiopia (1990–2000 vs. 1985–2000). Each analysis included three categories: all BMI groups, normal BMI (18.5–25 kg/m^2^), and high BMI (≥ 25 kg/m^2^). When the association was tested among Israeli-born men (**a**), the reference group for each analysis was native Israelis; and when it was tested for immigrant men (**b**), the reference group was USSR origin. Models were adjusted for BMI as a continuous variable in addition to the adjustments of the main analysis (see Table 2)

Point estimates for incident diabetes were directly related to the time interval from immigration. Point estimates were higher among those who immigrated to Israel before age 6 years (HR = 4.6, 95%CI = 2.6–8.2) than for those who immigrated after age 12 years (HR = 3.1, 95%CI = 1.6–5.9) (Additional file 1: Table S3). Also, the HR for diabetes among Israelis who were born in Ethiopia was lower than among second-generation Israelis of Ethiopian origin – 2.8 (2.0–3.9) vs. 4.0 (2.2–7.3) (native Israelis were the reference group; the model was adjusted for birth year, age at study entry, education level, cognitive score, and BMI (continuous); Additional file 1: Table S3). When the analysis was limited to men of Ethiopian origin who were born in Israel, the HRs were 4.4 (1.7–11.4) in the normal BMI group and 29.1 (12.0–70.6) in the high BMI group compared to native Israelis with normal BMI (Table 2). In contrast, risk for diabetes was comparable among Israeli-born men of USSR origin to that of native Israelis at any BMI stratum (Fig. 2a). For immigrants from Ethiopia, the adjusted HR to develop type 2 diabetes was 3.9 (2.8–5.5) compared to immigrants from the USSR (Fig. 2b). This risk was more pronounced among immigrants from Ethiopia with normal BMI at study entry [HR = 5.1 (3.4–7.8)] when immigrants from the USSR with normal BMI served as the reference (Fig. 2b).

The results were accentuated when logistic regression models were applied, and with the inclusion of incident cases of type 2 diabetes for whom the date of diagnosis was missing (n = 64; Additional file 1: Table S1). Odds ratios (ORs) were calculated for the development of type 2 diabetes, using native Israelis with normal BMI as the reference group. The adjusted odds ratio (OR) for Israelis of Ethiopian origin with normal BMI was 4.1 (2.9–5.9). Among individuals with high BMI, the OR for those of Ethiopian origin was 18.8 (10.5–33.6), and for native Israelis 7.4 (5.4–10.2); this difference between the culture groups was statistically significant (p = 0.028). Accounting for misclassification of diabetes diagnosis, either by including cases of uncertain diabetes type or by applying a stricter definition of type 2 diabetes with over 98% positive predicted value, yielded similar results (Additional file 1: Table S2).

### Incident type 2 diabetes among Israeli women of Ethiopian Origin

During 530,814 person-years there were 79 cases of incident type 2 diabetes among women: 29 (0.27%) in those of Ethiopian origin and 50 (0.15%) in native Israelis. The mean ages at diabetes diagnosis were 28.3 and 27.3 years, respectively. Notably, high BMI among female adolescents of Ethiopian origin was rare, only 6 persons, in the first decade of the study. The crude incident rate for type 2 diabetes (per 10^5^ person-years) in Ethiopian vs. native Israeli women were 10.5 vs. 0 in the underweight group, 23.7 vs. 5.7 in the normal BMI group, and 54.0 vs. 55.8 in the high BMI group (Table 2). These differences were well apparent in Kaplan–Meier survival analysis (Fig. 1b, d, f). The BMI recorded at the time of entrance to the INDR had no consistent pattern. Women of Ethiopian origin who developed type 2 diabetes in the normal BMI group gained more weight than did their native counterparts, whereas the opposite was observed for the high BMI group (Additional file 1: Figure S2B). Among women with normal BMI in adolescence, the HR for type 2 diabetes was 4.2 (2.2–8.1) for those of Ethiopian origin compared to their native peers; and 2.4 (1.1–5.1) in the adjusted model. The latter was attenuated to borderline significance when adjusted logistic regression models were applied to include incident cases without the date of diabetes diagnosis (n = 47; OR = 1.8, 95%CI 0.98–3.2, p = 0.058; Additional file 1: Table S1). HRs of Ethiopian and native Israeli women with high adolescent BMI were comparable.

## Discussion

The main findings of this nationwide longitudinal study include the following: (1) For Israeli adolescent males of Ethiopian origin with normal or high BMI, the risk for incident early-onset diabetes is 3- to 4-fold higher than for native Israeli males. This difference is unlikely to be accounted by differential weight gain in adulthood and was not observed among immigrants to Israel from the former USSR countries, who arrived to Israel at the same period as immigration from Ethiopia. (2) Diabetes risk is accentuated in second-generation Israeli-born men of Ethiopian origin. Point estimates were stable following adjustment to sociodemographic background and extensive sensitivity analyses.

A solid body of evidence indicates an association of black ancestry with increased diabetes risk compared to white Caucasians. Examples are the disproportionate increased risk for early-onset diabetes among persons of African origin in the UK [21–23], Canada [24], and the US [25]. HRs for developing type 2 diabetes for middle-aged African Americans [2, 26–30] and Ethiopian immigrants to Israel [31, 32], compared to native populations, were reported in the range of 1.3–2.9. Among young adults and adolescents, point estimates ranged between 2.1 [30] and 6.2 [33, 34]. Methodological differences relating to the nature of population selection, the level of adjustment to confounders, and the wide age range at study entry may account for the differences observed. Additionally, in several US studies, African Americans were aggregated into a homogenous group despite diversity in ethnic and cultural background [11]. In this respect, our study is population-based, with a narrow age range at study entry, and a homogenous ethnic origin. In addition, a series of sociodemographic variables were included that were systematically obtained without a selection bias, given the mandatory nature of the medical screening.

Repeated evidence supports an association between genetic polymorphism and diabetes risk among persons of African ancestry. The thrifty gene hypothesis [35] suggests that certain populations have alleles that are adaptive to the feast and famine cycles of paleolithic human existence. These may promote rapid weight gain that may be advantageous during times of limited food availability but may be metabolically deleterious at times of continuing affluence. In agreement, several examples of novel and African ancestry-specific disease loci have been discovered in association with insulin resistance and inflammation. For example, polymorphisms in tumor necrosis factor-alpha (TNF-α) [36], or the anti-inflammatory adipokine, adiponectin [37], were more prevalent among persons of African ancestry and were linked to an increased risk for type 2 diabetes. Notably, young African Americans, who largely represent West African ancestry, have been shown to have an increased risk to develop diabetes regardless of BMI [30], similar to Israelis of Ethiopian origin. This suggests that the increased genetic predisposition for type 2 diabetes risk is largely driven by Pan-African ancestry. This contrasts with the genetic predisposition for end-stage renal disease that affects people of West African, but not Ethiopian origin [38].

We report higher risk for type 2 diabetes among second-generation Israeli-born Ethiopian men than among those who immigrated; this association correlated with the time interval from immigration. Our findings corroborate previous evidence that diabetes risk correlates with the time since immigration among immigrants from various ethnic backgrounds to Western countries [39, 40], and is higher among second- than first-generation immigrants by 2- to 6-fold [39, 41]. Similarly, African immigrants to the US were shown to be more likely to develop pre-diabetes or diabetes than African Americans, despite their having a higher level of education and lower BMI [8]. Notably, no association was found between diabetes incidence and immigration status regarding immigration from the former USSR, which occurred in parallel to the Ethiopian immigration. Additionally, previous studies underscored lifestyle changes among Ethiopian immigrants to Israel, who changed their diet to a more Western one [42], and became less physically active and with more obesity [19] in a manner that correlated with the time since immigration [43].

Our findings have implications to other high-income countries that are recipients of immigrants from Ethiopia. In the US, evidence supports the need to disaggregate the African American category into its ethnic subcommunities based on differences in genetic admixture, culture, and metabolic risk [8, 9, 44]. Of note, as of 2018, the US population included over 730,000 immigrants from East Africa. Nearly half of them were from Ethiopia, and they are considered to be the fastest-growing sub-African community in the US. This trend is more evident in some European countries such as Germany, where African immigrants constitute nearly 1% of the population [45]. The observation that the prevalence of high BMI among Israeli-born Ethiopian male adolescents has tripled over the last decade and reached nearly 20% [19] echoes with US data [46], and predicts an increased burden of type 2 diabetes in young adults from this population. The lower prevalence of type 2 diabetes in Ethiopia, by over threefold, compared to Israel, the US, and most other Western countries, further emphasizes the contribution of immigration to diabetes risk at young ages in high-income countries [1]. Importantly, undiagnosed diabetes among young adults may be over tenfold more prevalent among blacks than whites, as exemplified in the US population [34]. Collectively, these data emphasize the importance of clinical awareness, education for healthy lifestyle, and reduction of other preventable risk factors, especially in regard to adolescent obesity, for Ethiopian and possibly other East African immigrants.

This study has several limitations. First, only a single BMI measurement, at study entry, was available; and early longitudinal measurements throughout childhood and later during adulthood were unavailable. Nevertheless, BMI measurements among those who developed type 2 diabetes support that differential excess weight gain among Israelis of Ethiopian origin could not explain the results; this concurs with previous reports [31]. Additionally, the time interval from medical assessment at late adolescence to the mean age of diabetes onset was approximately 10 years. This period has the potential to confound the results due to lifestyle manners of the participants such as diet or exercise, but also constitutes a reasonable time frame for initiating intervention strategies. Second, we lacked data regarding pre-diabetes and undiagnosed diabetes, which may be especially prominent in a young population. Third, information on lifestyle measures including dietary intake, physical activity, and smoking were not available to us. Fourth, we lacked baseline information regarding plasma glucose, cholesterol, and other measures of adiposity such as waist circumference. Of note, BMI is considered the preferable method for screening adolescents according to the U.S. Preventive Task Force [47], and our dataset was based on a systematic medical screening process that enabled mitigating confounding by coexisting morbidities. Finally, we are underpowered to discuss the association between BMI and type 2 diabetes among young women with high BMI due to the small number of incident cases. However, findings in women with normal BMI resembled those in men. The strengths of this study include its systematic data collection at a narrow age range, the absence of population selection bias, and linkage between two nationwide databases and outcome cases of type 2 diabetes that were predominantly diagnosed before age 30 years. The ability to compare the findings among second generation Ethiopian descent Israelis with those of immigrants from other regions to Israel in this large sample size is advantageous.

## Conclusions

Adolescent males of Ethiopian origin are at increased risk for early-onset type 2 diabetes even if their BMI is within the normal range; and those with overweight and obesity are at considerably greater risk. These findings emphasize the need for tight medical follow-up, aimed at reducing concomitant risk factors including obesity prevention in particular, education for healthy lifestyle, and early intervention medical therapy as needed.

## Supplementary information

**Additional file 1: Appendix 1.** Extended methods. **Figure S1.** Schematic diagram of the study design and cohort build-up. **Figure S2.** BMI at entrance to the Israeli National Diabetes Registry by adolescent BMI categories. **Table S1.** Accounting for incident type 2 diabetes cases with missing date of diabetes onset- Logistic regression models. **Table S2.** Accounting for misclassification in the diagnosis of type 2 diabetes among men. **Table S3.** Type 2 diabetes among men of Ethiopian origin, first and second-generation immigrants to Israel.

## Data Availability

Data sharing is not applicable for this study.

## Abbreviations

BMI: Body Mass Index
CI: Confidence Interval
HR: Hazard Ratio
SD: Standard Deviation
SES: Socio-economic Status
USSR: Union of Soviet Socialist Republics
