# Supplementary material for: Adolescent BMI and early-onset type 2 diabetes among Ethiopian immigrants and their descendants: a nationwide study
Source: Cardiovasc Diabetol. 2020 Oct 6;19:168. doi: 10.1186/s12933-020-01143-z (PMC7542395; doi:10.1186/s12933-020-01143-z)
Supplement: Supplementary file 1 — Additional file 1: Appendix 1. Extended methods. Figure S1. Schematic diagram of the study design and cohort build-up. Figure S2. BMI at entrance to the Israeli National Diabetes Registry by adolescent BMI categories. Table S1. Accounting for incident type 2 diabetes cases with missing date of diabetes onset- Logistic regression models. Table S2. Accounting for misclassification in the diagnosis of type 2 diabetes among men. Table S3. Type 2 diabetes among men of Ethiopian origin, first and second-generation immigrants to Israel. [file 12933_2020_1143_MOESM1_ESM.docx]

**Supplementary Appendix Table of Contents**

| **Content** | **Description** | **Page** |
| --- | --- | --- |
| Appendix 1 | Extended methods. | 2 |
| Figure S1 | Schematic diagram of the study design and cohort build-up. | 3 |
| Figure S2 | BMI at entrance to the Israeli National Diabetes Registry by adolescent BMI categories. | 4 |
| Table S1 | Accounting for incident type 2 diabetes cases with missing date of diabetes onset- Logistic regression models. | 6 |
| Table S2 | Accounting for misclassification in the diagnosis of type 2 diabetes among men. | 7 |
| Table S3 | Type 2 diabetes among men of Ethiopian origin, first and second-generation immigrants to Israel. | 8 |

**Appendix 1: Extended Methods.**

Classification to type 1, type 2, and uncertain type of diabetes:

We excluded from the outcome of type 2 diabetes individuals who were classified as having type 1 diabetes by the following criteria: (i) treatment with short-acting insulin was initiated within one year of diabetes onset, or (ii) insulin treatment was documented without prior evidence of treatment with oral anti-diabetic drugs. We recorded all other incidences of diabetes as type 2 diabetes. Diabetes of uncertain type was the classification used when information on ant-diabetes medications was missing. Gestational diabetes is not reported to the Israeli National Diabetes Registry and were therefore not included in this study.

Categorization of education and residential socioeconomic status:

Education level was categorized as low (<11 years) and high (>11 full years of schooling). Socioeconomic status (SES) was based on residential locality and was categorized on a scale of 1-10 based on the Israeli Central Bureau of Statistics scoring system^(1)^. This score stratifies all municipalities into 10 decile groups considering multiple variables that might affect SES. We grouped the scores to low (SES=1^st^-4^th^ decile), medium (SES=5^th^-7^th^ decile), and high (SES=8^th^-10^th^ decile).

References:

1. Statistics ICB of. Characterization and classification of local authorities by the socio-economic level of the population. 2006.

**Supplementary Figure S1: Schematic diagram of the study design and cohort build-up.**

***Pre-recruitment evaluation during 1996-2011 (N=1,119,049) of adolescents, aged 16-20 years.***

*Medical assessment*

Review of health summary by examinees' family physicians.

Detailed medical interview and physical examination by a physician.

Anthropometric measurement.

*Collection of sociodemographic data*

Years of education.

Residential socioeconomic status.

Cognitive test.

***Excluded (N=1,023,691)***

Adolescents of USSR origin*

***Origin:***

Native Israelis (3^rd^ generation in Israel).

Israelis of Ethiopian origin

***N= 125,358*** *(men=80,970; women=44,388)*

***Israelis N=93,806***

Men=60,771

Women=33,035

***Ethiopians N=27,684***

Men=17,050

Women=10,634

***121,490 participants***

Men=77,821

Women=43,669

***Excluded (N=3,868)***

Deaths before 2011 (n=378).

History of diabetes or dysglycemia (n=325).

Missing BMI data (n=3,291).

*Of the 1,023,691 persons who were not native Israelis or of Ethiopian origin, we included in a sub-analysis 121,997 individuals (see Figure 2).

**Supplementary figure S2:** **BMI at entrance to the Israeli National Diabetes Registry (INDR) by adolescent BMI categories.** Mean BMI at study entry and at the year of diagnosis in the INDR among men (A) and women (B). The results were stratified by adolescent BMI status. Significant differences were found between all the categories at study entry and between the INDR and the study entry values for men with normal BMI. Red bars refer to Israelis of Ethiopian origin and blue bars refer to native Israelis.

Study entry INDR Study entry INDR Study entry INDR

**Underweight Normal High**

**Adolescent BMI**

**(A) Men**

**(B) Women**

Study entry INDR Study entry INDR Study entry INDR

**Underweight Normal High**

**Adolescent BMI**

**Supplementary Table S1: Accounting for incidences of type 2 diabetes with missing date of diabetes onset- Logistic regression models.** We applied logistic regression models to analyze incidences of type 2 diabetes with missing date of diabetes diagnosis. The analysis included an additional 64 men and 47 women who were excluded from the main analysis (Table 2). The model was the same as in Table 2, using native Israelis with normal BMI as the reference. Adolescent BMI units are kg/m^2^.

|  | **Native Israelis** | | | **Ethiopian Israelis** | | |
| --- | --- | --- | --- | --- | --- | --- |
|  | **Underweight** | **Normal BMI** | **High BMI** | **Underweight** | **Normal BMI** | **High BMI** |
| **Men** | | | | | | |
| Number of diabetes incidences | 6 | 63 | 101 | 16 | 87 | 17 |
| OR (unadjusted) | 0.55 | reference | 6.41 | 2.04 | 5.53 | 11.28 |
| 95% CI | 0.24-1.26 |  | 4.68-8.79 | 1.18-3.54 | 4.00-7.65 | 6.58-19.33 |
| p-value | 0.16 |  | 7.5×10^-31^ | 0.01 | 5.9×10^-25^ | 1.3×10^-18^ |
| OR (adjusted for birth year) | 0.50 | reference | 7.77 | 1.87 | 5.08 | 23.57 |
| 95% CI | 0.21-1.15 |  | 5.66-10.67 | 1.08-3.24 | 3.67-7.04 | 13.55-41.00 |
| p-value | 0.10 |  | 9.0×10^-37^ | 0.03 | 1.6×10^-22^ | 4.8×10^-29^ |
| OR (fully adjusted) | 0.52 | reference | 7.39 | 1.44 | 4.13 | 18.82 |
| 95% CI | 0.22-1.19 |  | 5.35-10.19 | 0.81-2.57 | 2.90-5.88 | 10.53-33.64 |
| p-value | 0.12 |  | 3.8×10^-34^ | 0.22 | 4.2×10^-15^ | 3.8×10^-23^ |
| **Women** | | | | | | |
| Number of diabetes incidences | 3 | 34 | 51 | 5 | 27 | 6 |
| OR (unadjusted) | 0.44 | reference | 6.77 | 1.09 | 2.84 | 4.05 |
| 95% CI | 0.13-1.42 |  | 4.38-10.45 | 0.43-2.80 | 1.71-4.70 | 1.70-9.68 |
| p-value | 0.17 |  | 7.0×10^-18^ | 0.85 | 5.4×10^-5^ | 1.6×10^-3^ |
| OR (adjusted for birth year) | 0.42 | reference | 7.26 | 1.05 | 2.64 | 5.07 |
| 95% CI | 0.13-1.37 |  | 4.69-11.23 | 0.41-2.69 | 1.59-4.38 | 2.12-12.14 |
| p-value | 0.15 |  | 5.2×10^-19^ | 0.92 | 1.7×10^-4^ | 2.7×10^-4^ |
| OR (fully adjusted) | 0.41 | reference | 6.95 | 0.75 | 1.76 | 3.69 |
| 95% CI | 0.13-1.35 |  | 4.48-10.77 | 0.28-2.00 | 0.98-3.15 | 1.48-9.16 |
| p-value | 0.14 |  | 4.1×10^-18^ | 0.57 | 0.06 | 5.0×10^-3^ |

**Supplementary Table S2: Accounting for misclassification in the diagnosis of type 2 diabetes among men.** Several possibilities of diabetes misclassification were considered. We analyzed incidences of unknown type of diabetes and used a stricter definition of type 2 diabetes (incidences that were reported to the Israel National Diabetes Registry at least twice, specificity 98.2%). To account for misclassification of type 1 diabetes as type 2, we narrowed the outcome to incidences of type 2 diabetes that were not treated with insulin. Results of the main analysis from Table 2 were copied to facilitate comparison. All the models were adjusted as in Table 2. The reference group is native Israelis with BMI between 18.5 and 25 kg/m^2^.

|  | **Native Israelis** | | | **Ethiopian Israelis** | | |
| --- | --- | --- | --- | --- | --- | --- |
|  | **Underweight** | **Normal BMI** | **High BMI** | **Underweight** | **Normal BMI** | **High BMI** |
| Type 2 Diabetes (main analysis) | | | | | | |
| HR (adjusted) | 0.52 | reference | 6.93 | 1.18 | 3.41 | 15.83 |
| 95% CI | 0.21-1.31 |  | 4.86-9.88 | 0.60-2.29 | 2.29-5.06 | 8.29-30.25 |
| p-value | 0.17 |  | 1.3×10^-26^ | 0.63 | 1.4×10^-9^ | 6.3×10^-17^ |
| Type 2 and unknown type of diabetes | | | | | | |
| HR (adjusted) | 0.59 | reference | 5.96 | 1.12 | 3.25 | 15.07 |
| 95% CI | 0.27-1.30 |  | 4.28-8.28 | 0.60-2.11 | 2.25-4.70 | 8.29-27.40 |
| p-value | 0.19 |  | 2.7×10^-26^ | 0.72 | 3.7×10^-10^ | 6.0×10^-19^ |
| Strict definition of type 2 diabetes | | | | | | |
| HR (adjusted) | 0.73 | reference | 9.18 | 1.04 | 3.27 | 11.33 |
| 95% CI | 0.26-2.08 |  | 5.87-14.36 | 0.42-2.54 | 1.95-5.49 | 4.30-29.82 |
| p-value | 0.56 |  | 2.5×10^-22^ | 0.94 | 7.4×10^-6^ | 8.9×10^-7^ |
| Type 2 diabetes not treated with insulin | | | | | | |
| Number of cases | 5 | 50 | 77 | 11 | 61 | 11 |
| HR (adjusted) | 0.54 | reference | 6.92 | 1.11 | 3.37 | 14.95 |
| 95% CI | 0.22-1.36 |  | 4.82-9.94 | 0.55-2.22 | 2.25-5.06 | 7.63-29.31 |
| p-value | 0.19 |  | 1.3×10^-25^ | 0.77 | 4.3×10^-9^ | 3.3×10^-15^ |

**Supplementary Table S3: Type 2 diabetes among men of Ethiopian origin, first and second-generation immigrants to Israel.** (A) The association between age at immigration from Ethiopia and the hazard ratio (HR) for incident type 2 diabetes. Of the 7,995 men who immigrated to Israel from Ethiopia and had normal adolescent BMI at study entry, 6,919 (87%) had normal BMI and documented immigration dates. These were grouped by age at immigration as shown. HRs for incident type 2 diabetes were computed using native Israelis as the reference group, with adjustment to variables as described in Table 2. Note that point estimates increased as the time interval from immigration increased. (B) The hazard ratio for incident type 2 diabetes among first (Ethiopian-born) and second-generation Ethiopian Israelis. The model was adjusted for birth year, age at study entry, education level, cognitive score, and BMI [continuous], with native Israelis as the reference group.

**(A) Men with normal BMI by age of immigration.**

|  |  | **Immigration age (years)** | | |
| --- | --- | --- | --- | --- |
|  | **Native Israelis** | **0-6** | **6-12** | **12-20** |
| Number of individuals | 42,166 | 2,632 | 2,635 | 1,652 |
| Number of type 2 diabetes incidences | 52 | 17 | 22 | 16 |
| HR (adjusted) | reference | 4.62 | 3.27 | 3.08 |
| 95% CI |  | 2.60-8.20 | 1.91-5.61 | 1.62-5.85 |
| p-value |  | 1.8×10^-7^ | 1.7×10^-5^ | 0.01 |

**(B) First vs. second generation men of Ethiopian origin and type 2 diabetes risk.**

|  | **Origin** | | |
| --- | --- | --- | --- |
|  | **Native Israelis** | **Ethiopian-born** | **Israeli-born Ethiopians** |
| Number of individuals | 60,771 | 12,080 | 4,970 |
| Number of type 2 diabetes incidences | 137 | 76 | 13 |
| Mean follow-up (years ± SD) | 12.8±4.6 | 14.1±4.5 | 9.9±3.3 |
| Cumulative follow-up (person-years) | 774,481.3 | 169,283.1 | 49,215.9 |
| Incident rate (per 10^5^ person-years) | 17.69 | 44.90 | 26.41 |
| HR (adjusted) | reference | 2.77 | 3.98 |
| 95% CI |  | 1.97-3.90 | 2.18-7.26 |
| p-value |  | <0.001 | <0.001 |
